# Supplementary material for: Two New Cytotoxic Steroidal Alkaloids from Sarcococca Hookeriana
Source: Molecules. 2018 Dec 20;24(1):11. doi: 10.3390/molecules24010011 (PMC6337136; doi:10.3390/molecules24010011)
Supplement: Supplementary file 1 [file molecules-24-00011-s001.pdf]

Supporting Information

Two new cytotoxic steroidal alkaloids from *Sarcococca hookeriana*

Shaojie Huo[1], Jichun Wu[1], Xicheng He[1], Lutai Pan[2] and Jiang Du[1, 2*]

[1] Guiyang College of Traditional Chinese Medicine, Guiyang 550025, China; 15038115764@163.com (S.H.); wujichun2018@sina.com (J.W.); hexicheng53@126.com (X.H.)

[2] Guizhou Provincial Key Laboratory of Miao Medicine, Guiyang 550025, China; ltpan@sina.cn (L.P.)

* Correspondence: dujang.gz@163.com (J.D.); Tel.: +86-871-88308060 (J.D.)

List of Contents

Fig.S1. UV Spectrum of Compound **1** 3

Fig.S2. IR Spectrum of Compound **1** 3

Fig.S3. HR-ESI-MS Spectrum of Compound **1** 4

Fig.S4. The ^{1}H NMR spectrum of compound **1** in $CDCl_3$ (500MHz) 4

Fig.S5. The ^{13}C NMR spectrum of compound **1** in $CDCl_3$ (125MHz) 5

Fig.S6. The DEPT spectrum of compound **1** in $CDCl_3$ (125MHz) 5

Fig.S7. The COSY spectrum of compound **1** in $CDCl_3$ (500MHz) 6

Fig.S8. The HSQC spectrum of compound **1** in $CDCl_3$ (500MHz) 6

Fig.S9. The HMBC spectrum of compound **1** in $CDCl_3$ (500MHz) 7

Fig.S10. The ROESY spectrum of compound **1** in $CDCl_3$ (500MHz) 7

Fig.S11. UV Spectrum of Compound **2** 8

Fig.S12. IR Spectrum of Compound **2** 8

Fig.S13. HR-ESI-MS Spectrum of Compound **2** 9

Fig.S14. The ^{1}H NMR spectrum of compound **2** in $CDCl_3$ (500MHz) 9

Fig.S15. The ^{13}CNMR spectrum of compound **2** in $CDCl_3$ (125MHz) 10

Fig.S16. The DEPT spectrum of compound **2** in $CDCl_3$ (125MHz) 10

Fig.S17. The COSY spectrum of compound **2** in $CDCl_3$ (500MHz) 11

Fig.S18. The HSQC spectrum of compound **2** in $CDCl_3$ (500MHz) 11

Fig.S19. The HMBC spectrum of compound **2** in $CDCl_3$ (500MHz) 12

Fig.S20. The ROESY spectrum of compound **2** in $CDCl_3$ (500MHz) 12

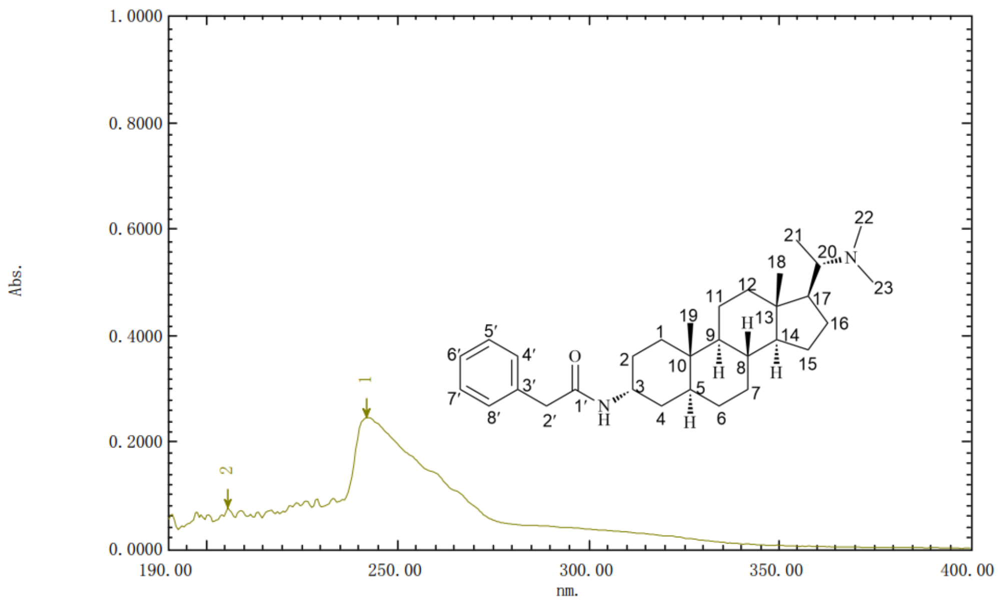

Fig.S1. UV Spectrum of Compound **1**

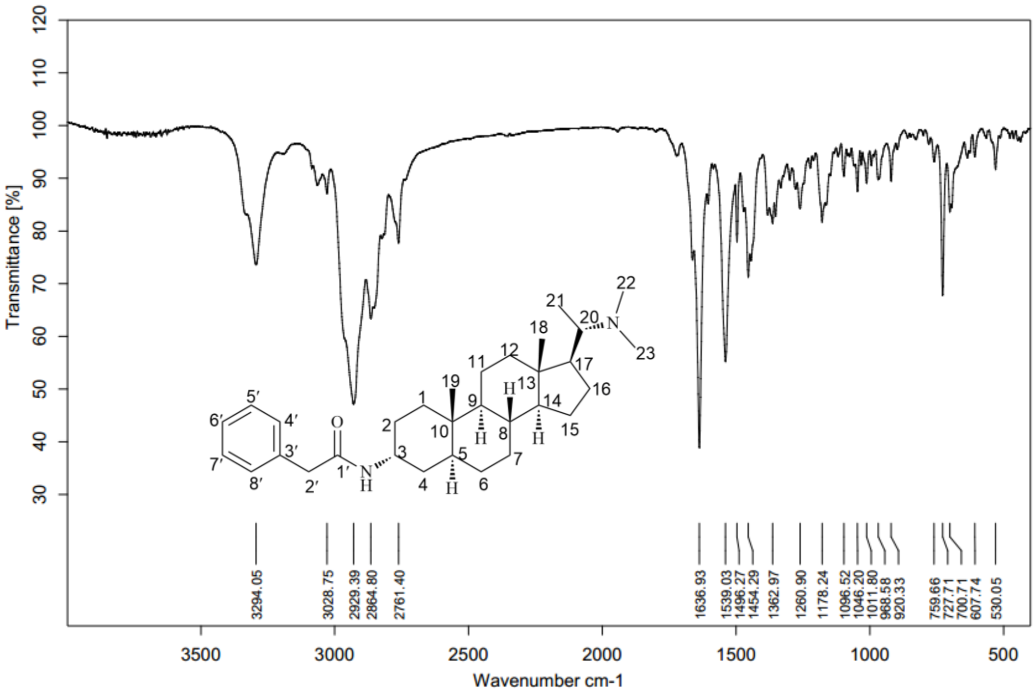

Fig.S2. IR Spectrum of Compound **1**

Mass Spectrum SmartFormula Report

Analysis Info

Analysis Name	D:\Data\lu yuan\ HS-25_8192.d	Acquisition Date	11/22/2017 5:20:48 PM
Method	tune low 20171012 pos.m	Operator	BDAL@DE
Sample Name	HS-25	Instrument	micrOTOF-Q II 228888.10354
Comment			

Acquisition Parameter

Source Type	ESI	Ion Polarity	Positive	Set Nebulizer	1.2 Bar
Focus	Active	Set Capillary	4500 V	Set Dry Heater	180 °C
Scan Begin	50 m/z	Set End Plate Offset	-500 V	Set Dry Gas	8.0 l/min
Scan End	1000 m/z	Set Collision Cell RF	400.0 Vpp	Set Divert Valve	Waste

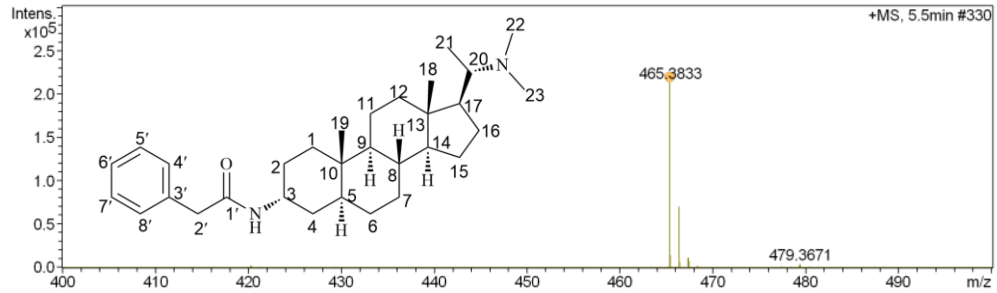

Meas. m/z	#	Ion Formula	m/z	err [ppm]	mSigma	# mSigma	Score	rdb	e⁻ Conf	N-Rule
465.3833	1	C31H49N2O	465.3839	1.4	9.6	1	100.00	8.5	even	ok

Fig.S3. HR-ESI-MS Spectrum of Compound **1**

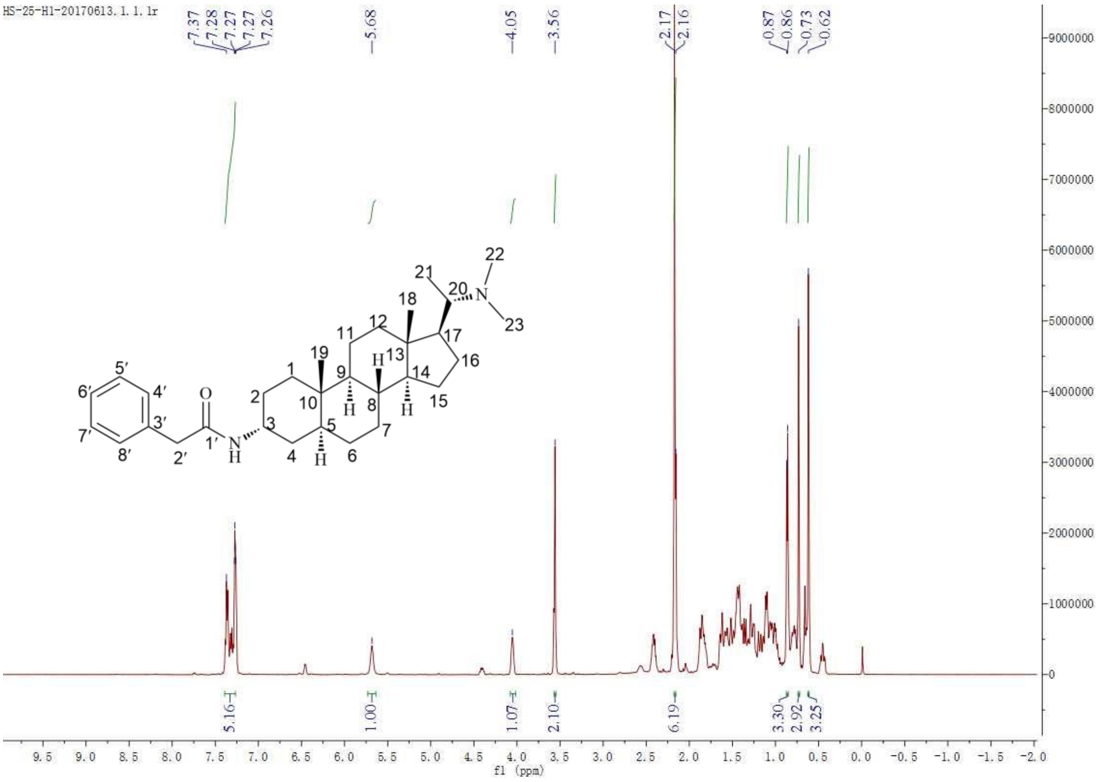

Fig.S4. The ^{1}H NMR spectrum of compound **1** in $CDCl_3$ (500MHz)

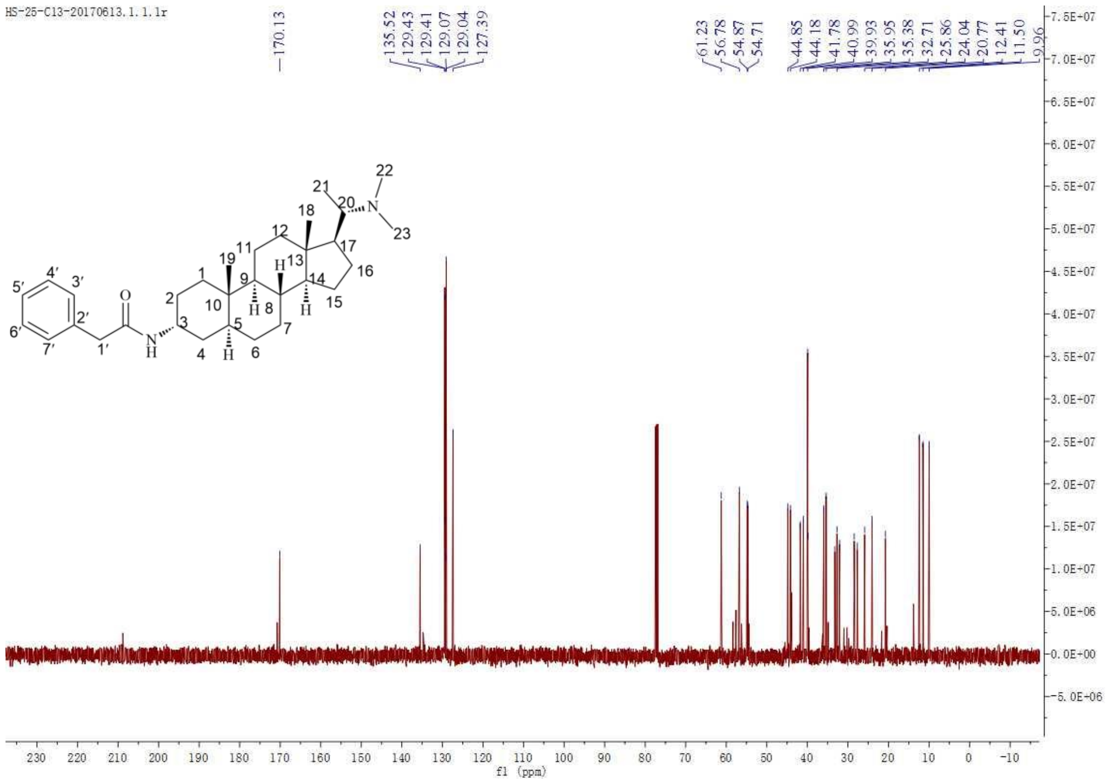

Fig.S5. The ^{13}C NMR spectrum of compound **1** in $CDCl_3$ (125MHz)

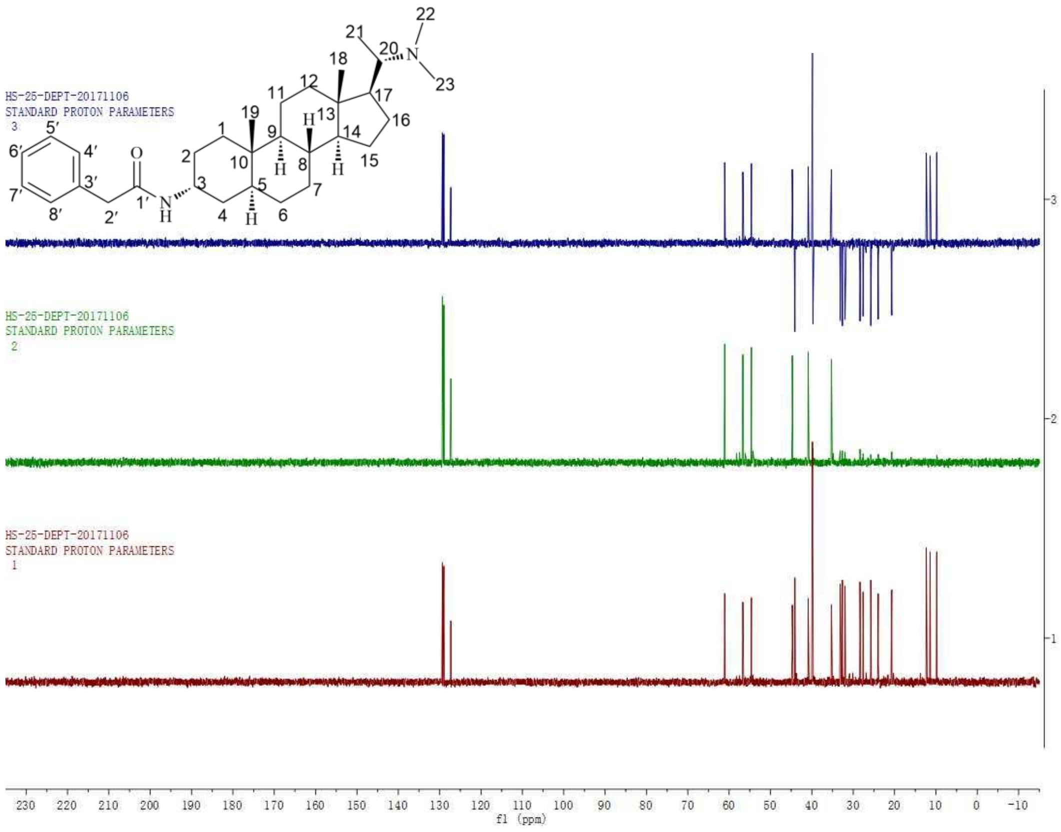

Fig.S6. The DEPT spectrum of compound **1** in $CDCl_3$ (125MHz)

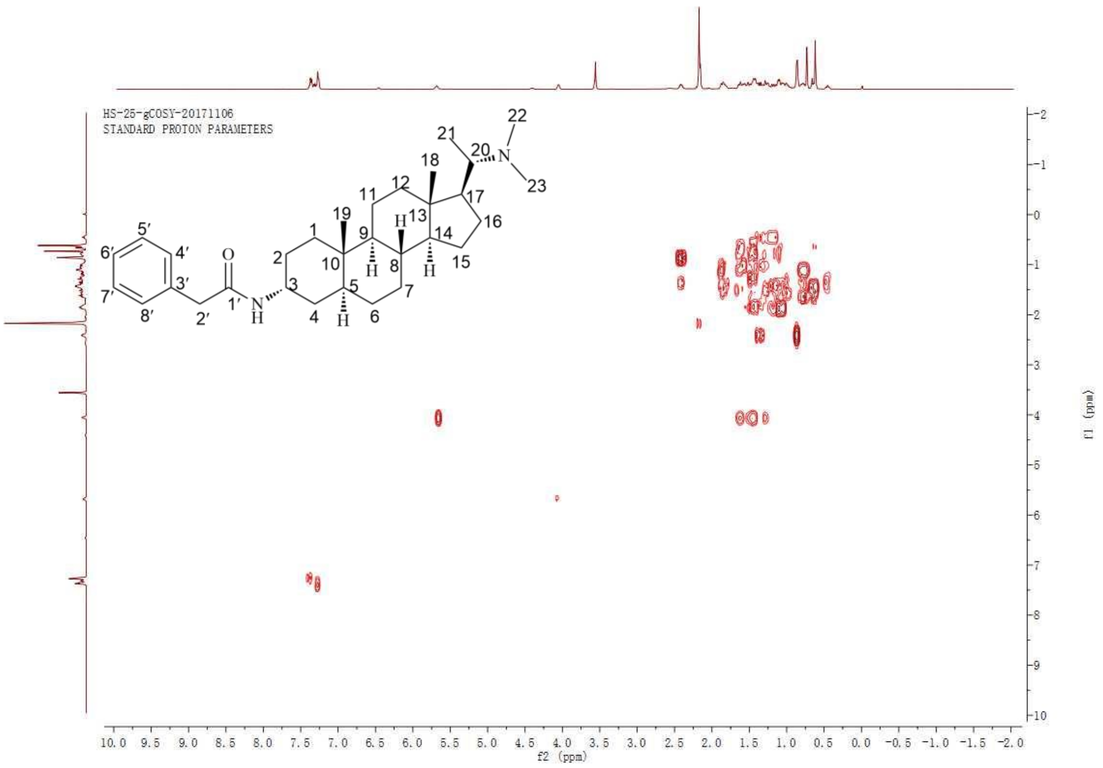

Fig.S7. The COSY spectrum of compound **1** in $CDCl_3$ (500MHz)

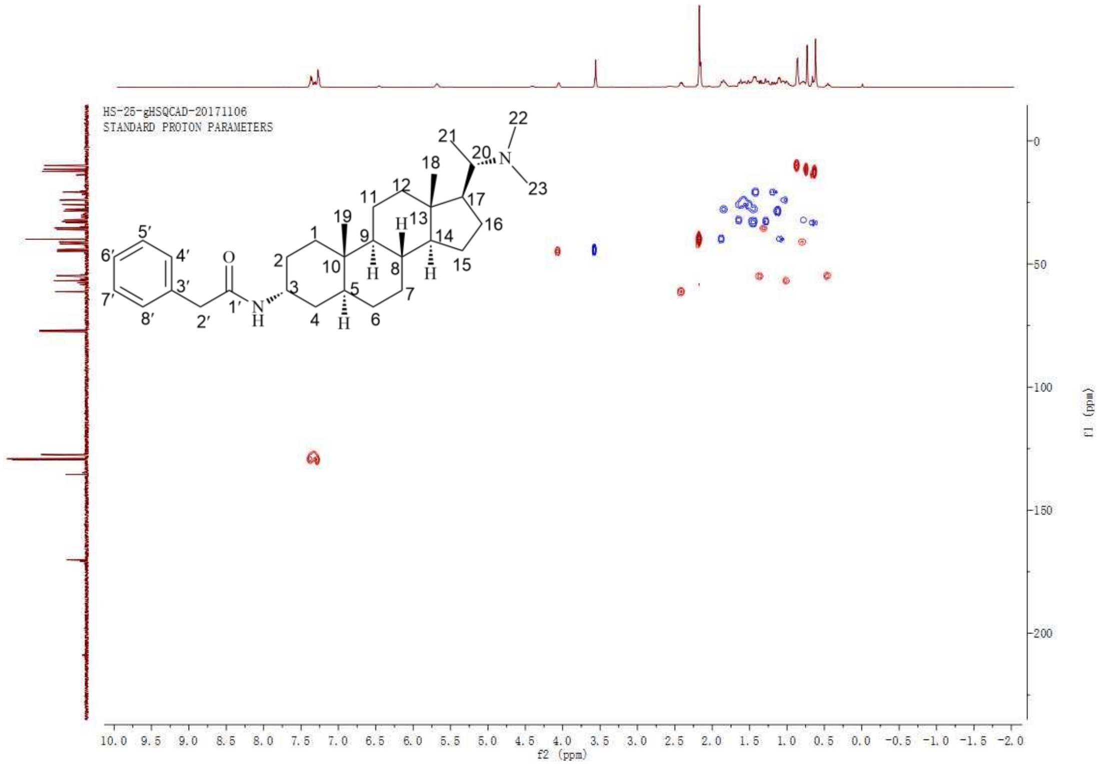

Fig.S8. The HSQC spectrum of compound **1** in $CDCl_3$ (500MHz)

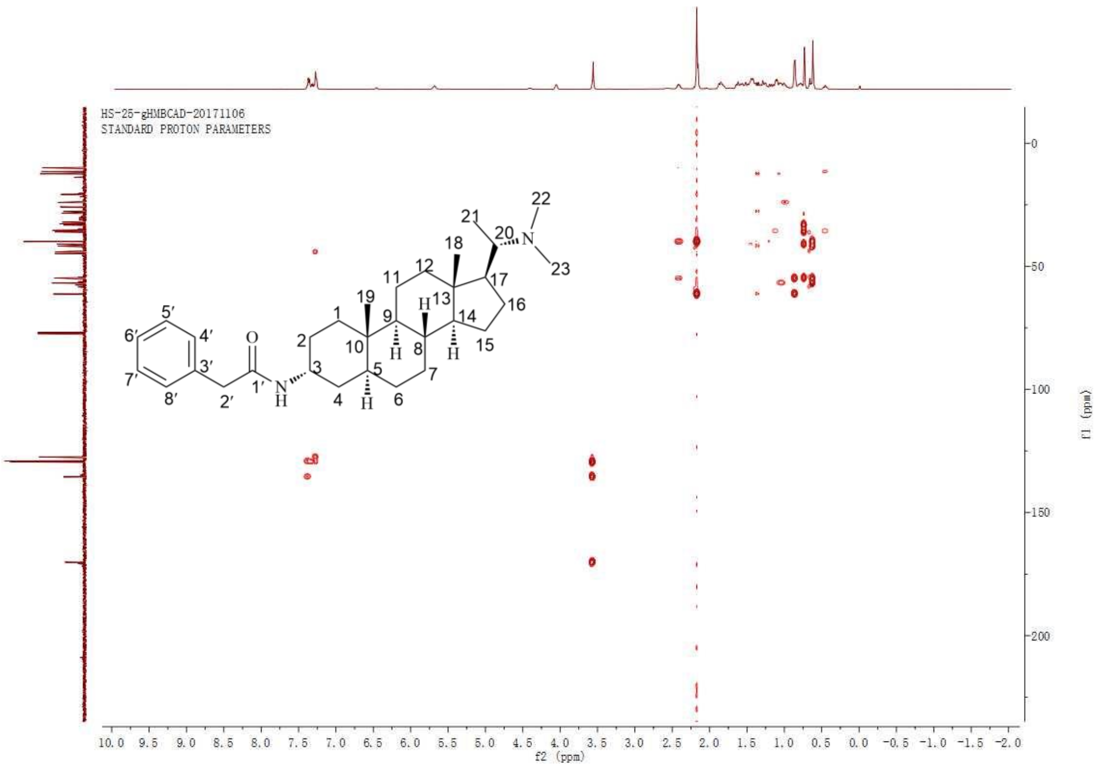

Fig.S9. The HMBC spectrum of compound **1** in $CDCl_3$ (500MHz)

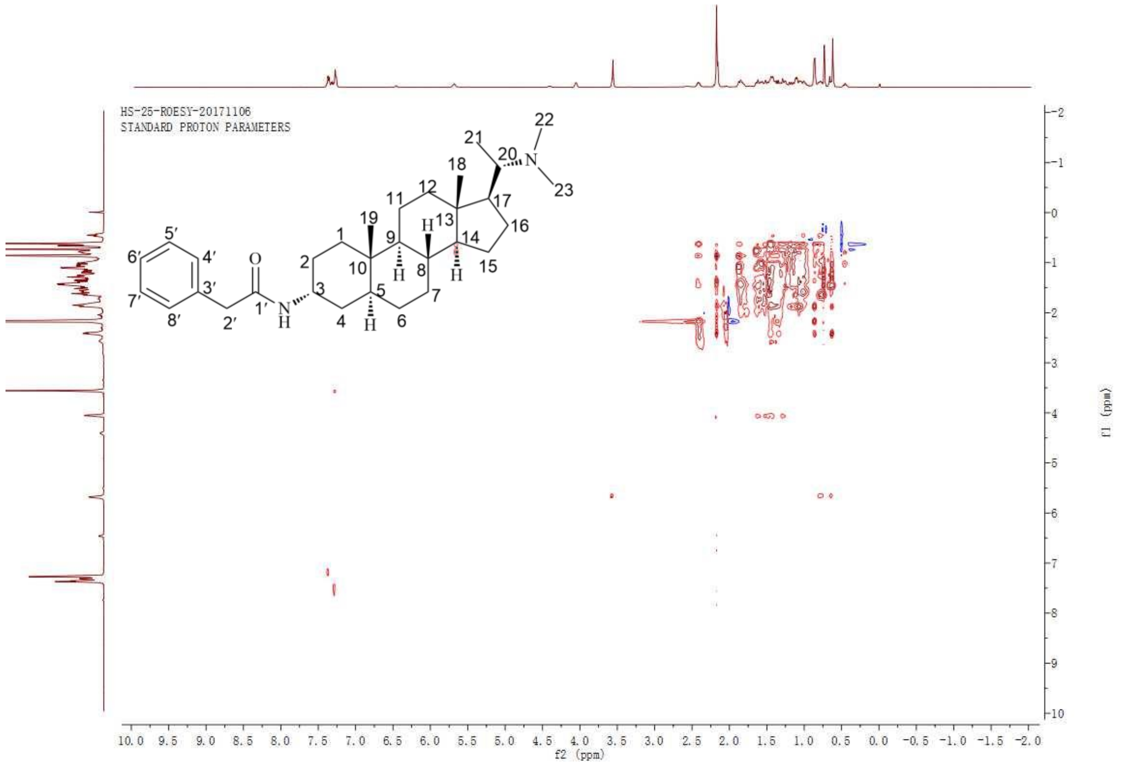

Fig.S10. The ROESY spectrum of compound **1** in $CDCl_3$ (500MHz)

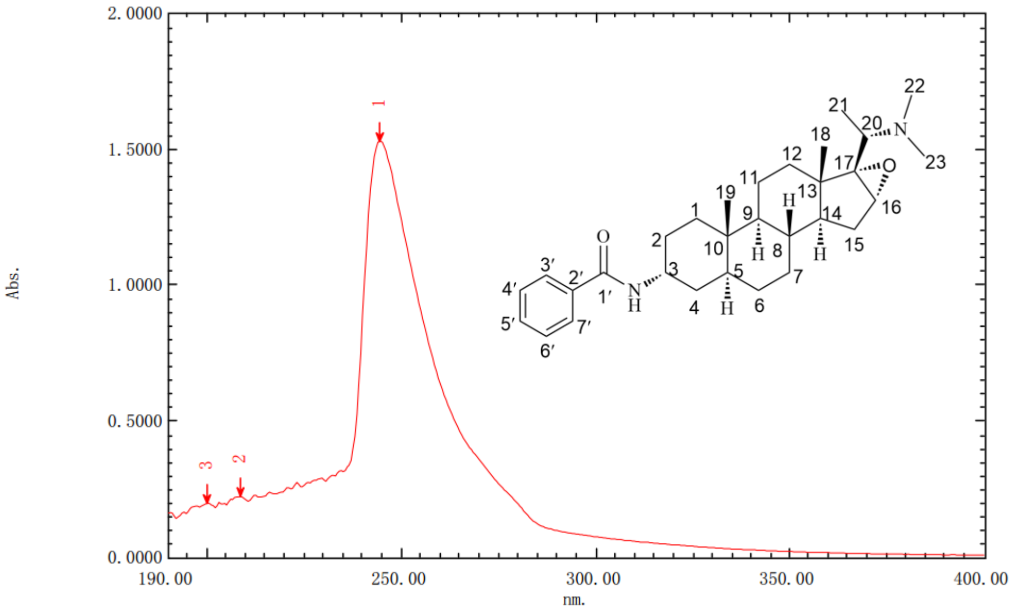

Fig.S11. UV Spectrum of Compound **2**

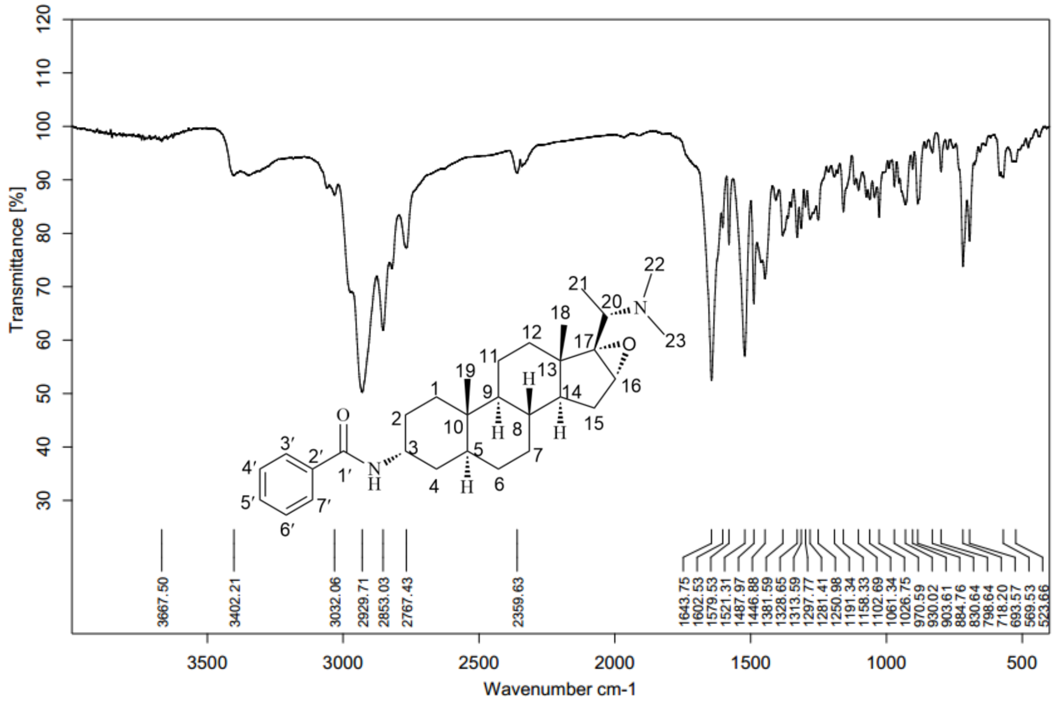

Fig.S12. IR Spectrum of Compound **2**

Mass Spectrum SmartFormula Report

Analysis Info

Analysis Name	D:\Data\lu yuan\ HS-26_8190.d	Acquisition Date	11/22/2017 4:57:22 PM
Method	tune low 20171012 pos.m		
Sample Name	HS-26	Operator	BDAL@DE
Comment		Instrument	micrOTOF-Q II 228888.10354

Acquisition Parameter

Source Type	ESI	Ion Polarity	Positive	Set Nebulizer	1.2 Bar
Focus	Active	Set Capillary	4500 V	Set Dry Heater	180 °C
Scan Begin	50 m/z	Set End Plate Offset	-500 V	Set Dry Gas	8.0 l/min
Scan End	1000 m/z	Set Collision Cell RF	400.0 Vpp	Set Divert Valve	Waste

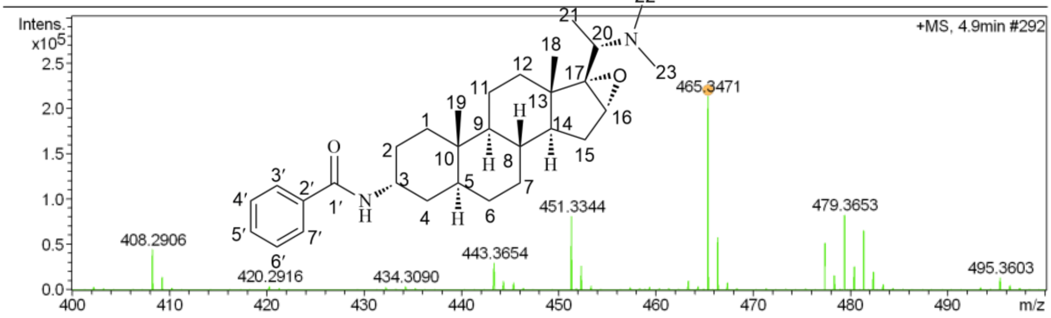

Meas. m/z	#	Ion Formula	m/z	err [ppm]	mSigma	# mSigma	Score	rdb	e⁻ Conf	N-Rule
465.3471	1	C30H45N2O2	465.3476	0.9	34.7	1	100.00	9.5	even	ok

Fig.S13. HR-ESI-MS Spectrum of Compound **2**

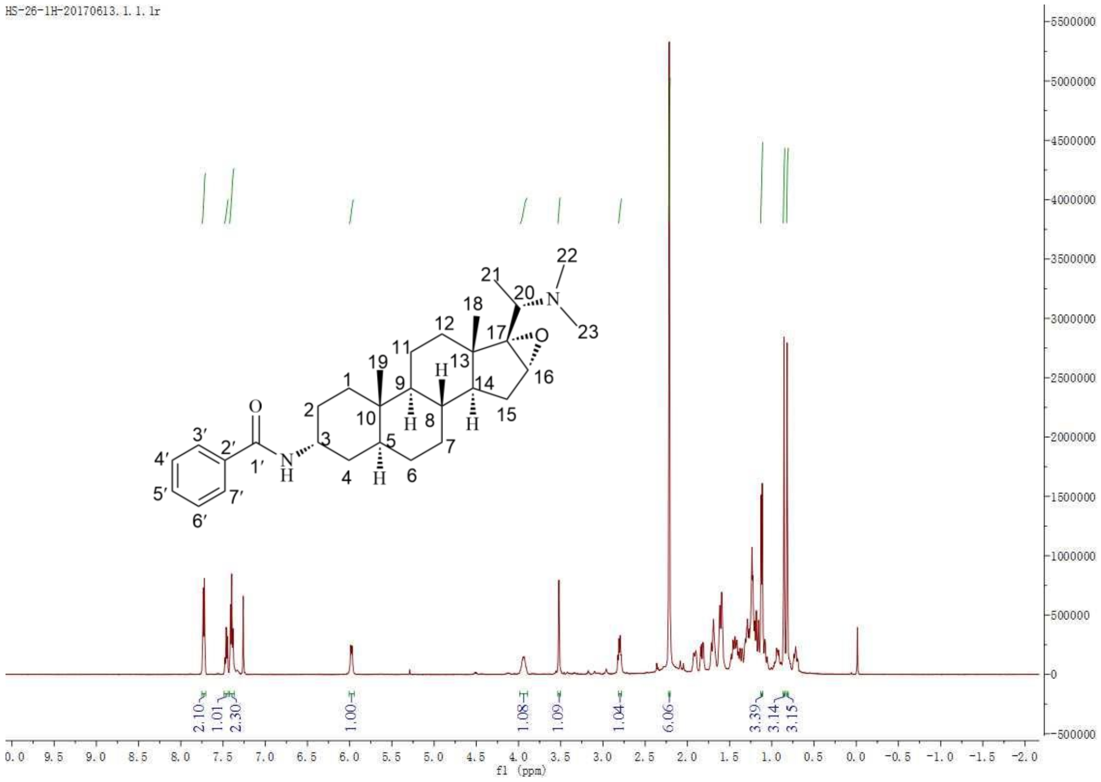

Fig.S14. The ^{1}H NMR spectrum of compound **2** in $CDCl_3$ (500MHz)

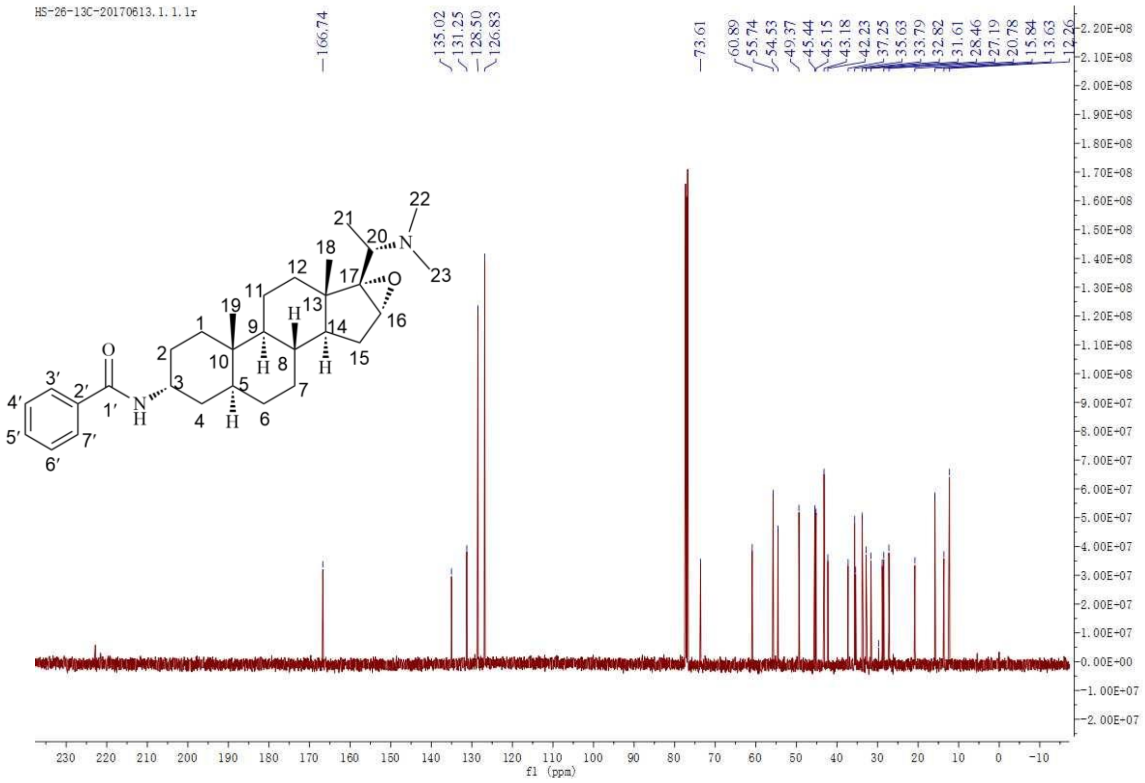

Fig.S15. The ^{13}CNMR spectrum of compound **2** in $CDCl_3$ (125MHz)

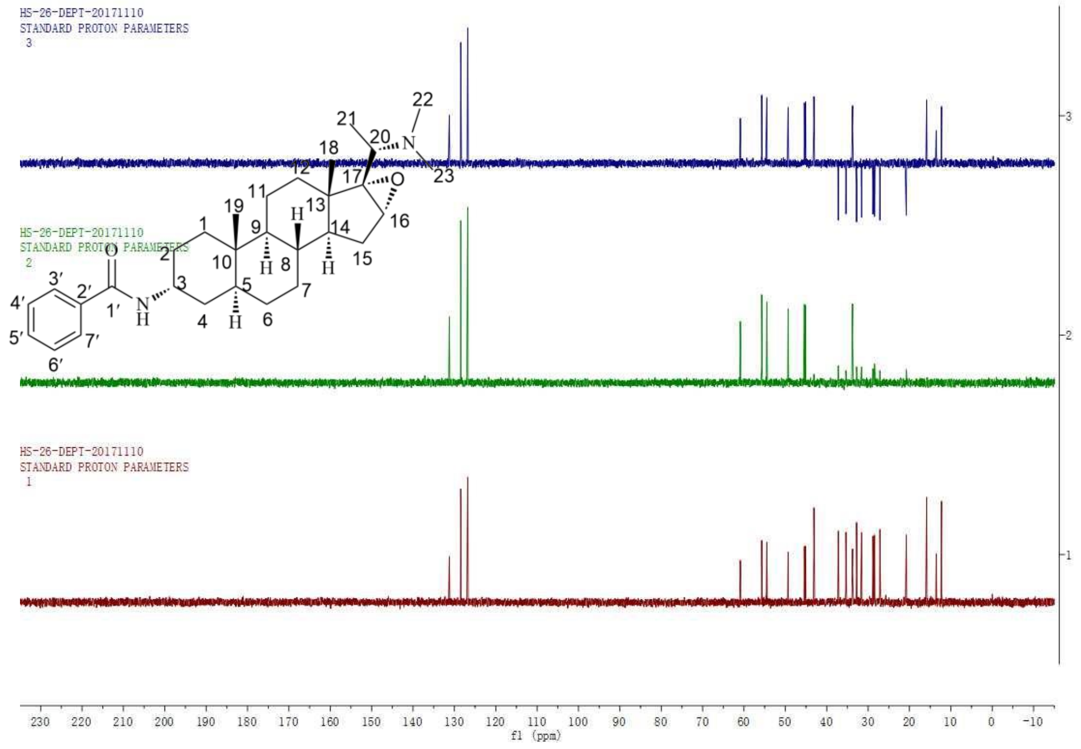

Fig.S16. The DEPT spectrum of compound **2** in $CDCl_3$ (125MHz)

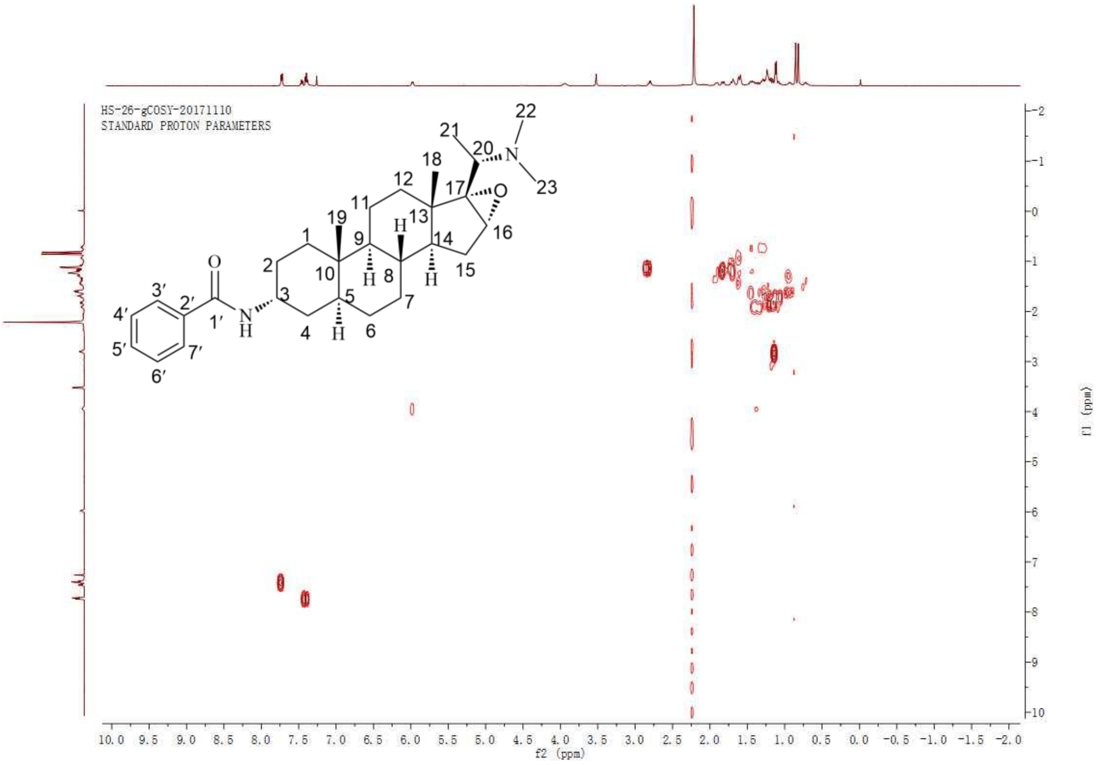

Fig.S17. The COSY spectrum of compound **2** in $CDCl_3$ (500MHz)

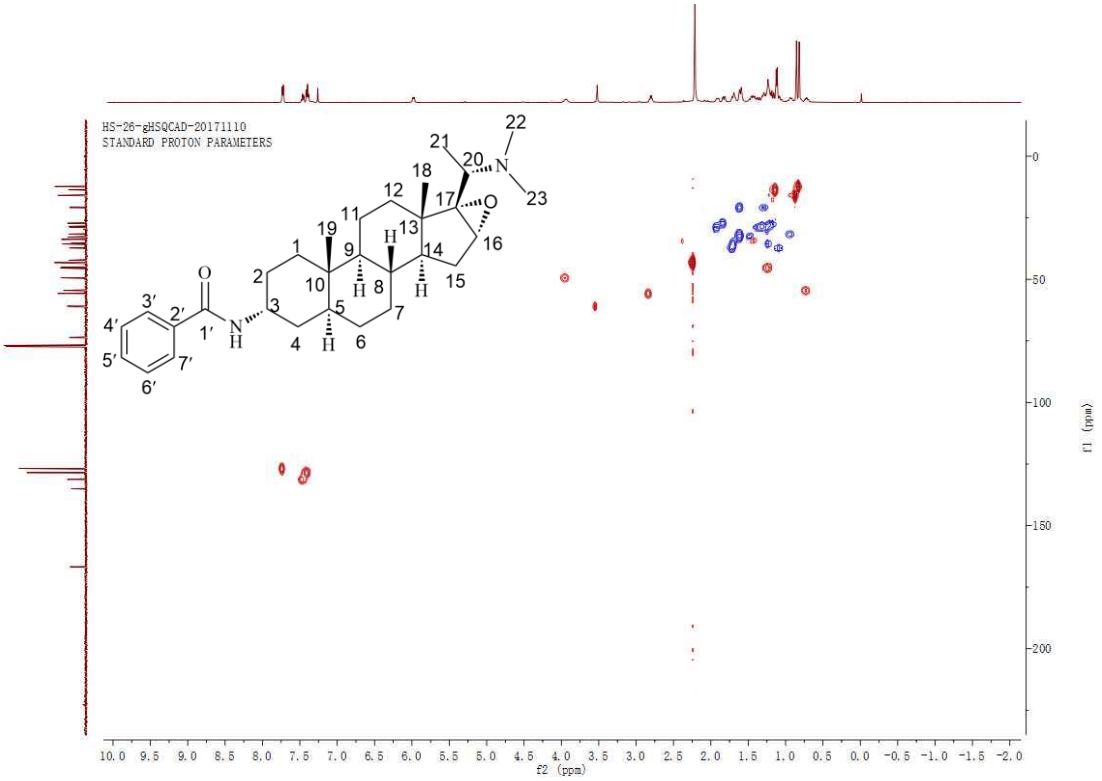

Fig.S18. The HSQC spectrum of compound **2** in $CDCl_3$ (500MHz)

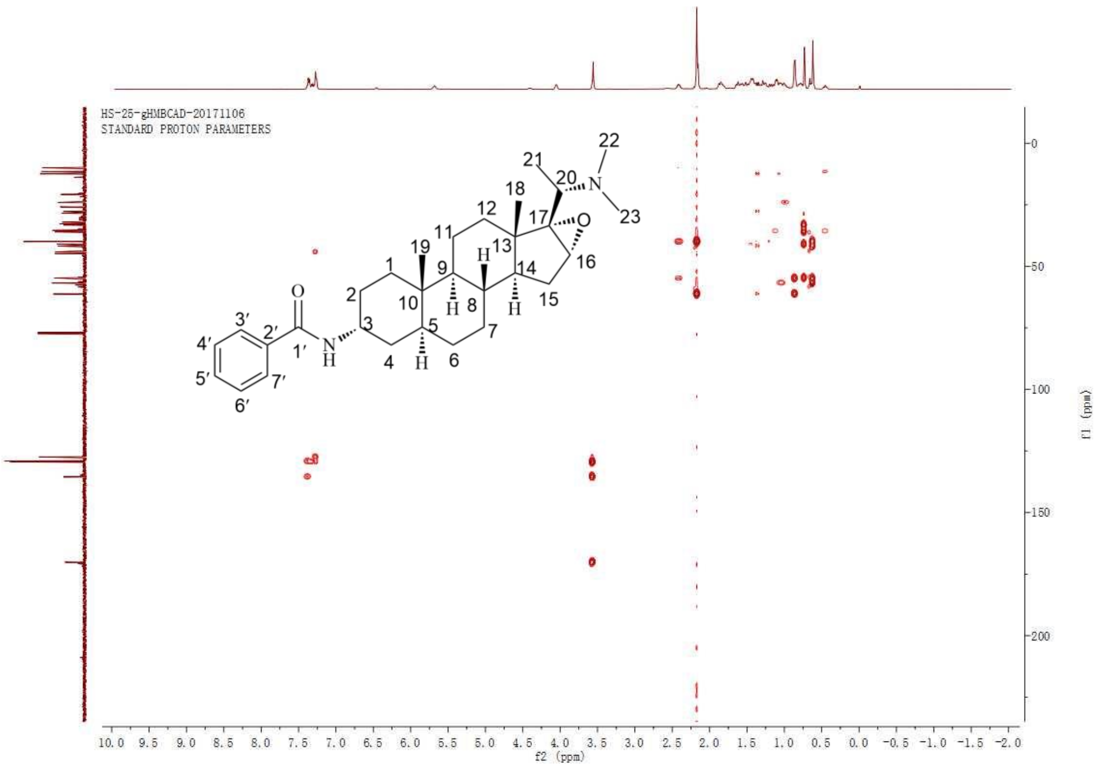

Fig.S19. The HMBC spectrum of compound **2** in $CDCl_3$ (500MHz)

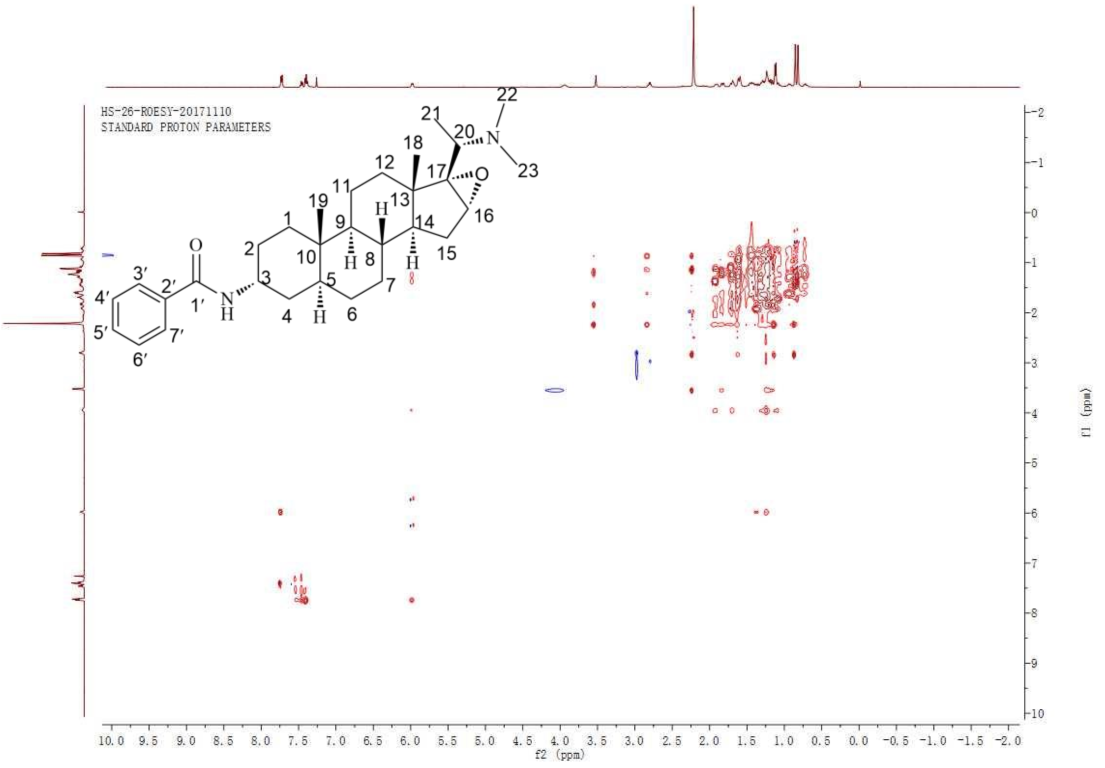

Fig.S20. The ROESY spectrum of compound **2** in $CDCl_3$ (500MHz)
